# QPHAR: quantitative pharmacophore activity relationship: method and validation

**DOI:** 10.1186/s13321-021-00537-9

**Published:** 2021-08-09

**Authors:** Stefan M. Kohlbacher, Thierry Langer, Thomas Seidel

**Affiliations:** Division of Pharmaceutical Chemistry, Department of Pharmaceutical Sciences, University of Vienna, Althanstraße 14, 1090 Vienna, Austria

**Keywords:** Pharmacophore, QSAR, Regression, Machine learning, Quantitative-pharmacophore-model

## Abstract

QSAR methods are widely applied in the drug discovery process, both in the hit‐to‐lead and lead optimization phase, as well as in the drug-approval process. Most QSAR algorithms are limited to using molecules as input and disregard pharmacophores or pharmacophoric features entirely. However, due to the high level of abstraction, pharmacophore representations provide some advantageous properties for building quantitative SAR models. The abstract depiction of molecular interactions avoids a bias towards overrepresented functional groups in small datasets. Furthermore, a well‐crafted quantitative pharmacophore model can generalise to underrepresented or even missing molecular features in the training set by using pharmacophoric interaction patterns only. This paper presents a novel method to construct quantitative pharmacophore models and demonstrates its applicability and robustness on more than 250 diverse datasets. fivefold cross-validation on these datasets with default settings yielded an average RMSE of 0.62, with an average standard deviation of 0.18. Additional cross-validation studies on datasets with 15–20 training samples showed that robust quantitative pharmacophore models could be obtained. These low requirements for dataset sizes render quantitative pharmacophores a viable go-tomethod for medicinal chemists, especially in the lead-optimisation stage of drug discovery projects.

**Supplementary Information:**

The online version contains supplementary material available at 10.1186/s13321-021-00537-9.

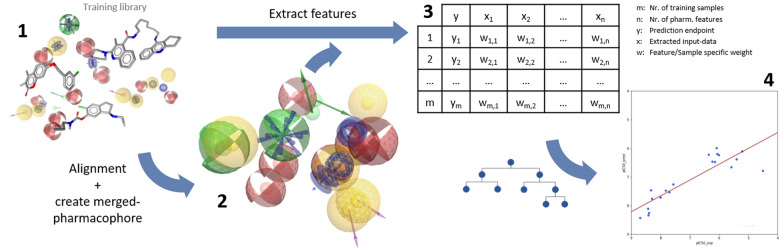

## Introduction

Quantitative structure–activity relationship (QSAR) studies were first introduced by Hansch et al. [1] in 1962 and have been growing in popularity ever since. Starting with simple correlations studies of chemical and biological properties, such as logP and K_i_ values, QSAR has evolved into a sophisticated method applying complex machine-learning (ML) models [2] on vast amounts of chemical data [3], often using more than a few thousand descriptors. QSAR models are not only useful for internal assistance in the drug discovery process, but highly validated and robust models have even been built by the FDA to assist the drug-approval process [4].

Over the years, QSAR has been influenced heavily by advanced machine learning [2] and other data processing systems, which effectively allows the researcher to extract more complex relationships from their data. With the use of more capable models, more complex input data can be processed. Countless descriptors or fingerprints [5] have been derived from 2D molecular structures but do not take into account spatial information and molecular conformation. Spatial information becomes even more critical when dealing with stereoisomers [6].

The popular QSAR modelling algorithm CoMFA [7], developed in the 80 s, uses 3D conformations of molecules as input, aligns them to each other, and then creates a predictive model from the molecules’ calculated steric and electrostatic interaction fields. The concept has gained wide popularity but never extended to different input domains than molecules. The method PHASE [8] proposed by Schrödinger [9] has taken this approach a step further. In addition to, or instead of calculating electrostatic interaction fields of the molecules, it is possible to generate pharmacophore fields from the input molecules. The same ML-algorithm as used in CoMFA, PLS (partial least squares), is then applied to create a predictive quantitative model. At the time, this has been a novelty since pharmacophores have only been used for qualitative virtual screening studies. Using pharmacophore fields derived from functional groups for quantitative modelling extends the CoMFA concept, using abstract 3D information of molecules for QSAR. Nevertheless, these pharmacophore fields are derived from molecules and a pure quantitative algorithm applied on pharmacophores has never been presented before.

Using pharmacophores as input in QSAR studies has several advantages: due to the abstract nature of pharmacophores, they are less influenced by small spatial perturbations of molecular features characteristic for such interactions. For example, bioisosteres are often highly similar in their interaction profile. They might cover, however, entirely different functional groups and substructures. Building a QSAR model on such data inevitably introduces a bias towards the predominant bioisosteric form occurring in the dataset. Pharmacophores, on the other hand, transform different functional groups with the same interaction profile into an abstract chemical feature representation associated with a particular non-bonding interaction type, such as a π-stacking interaction or H-Bond donor/acceptor interaction. This generalisation makes quantitative models more robust and less dependent on the dataset being used. Primarily in biological assays, robust predictive models are essential to avoid modelling the experimental noise [10].

Virtual screening takes advantage of pharmacophores’ abstract nature to achieve an effect known as “scaffold-hopping” [11]. Here, pharmacophores help to overcome a structural molecular bias by only considering the interaction patterns but not the molecular structures. A carefully constructed quantitative pharmacophore model will build on these advantages and the scaffold-hopping ability to harness its strengths. Besides abstracting molecular structures, pharmacophores also abstract the exact steric location and orientation of interactions by introducing tolerance ranges. Losing information on the precise position of possible interactions might not be desired with highly conserved protein targets. In general, however, generalisation is considered positive and avoids overfitted models.

Pharmacophore modelling is often used in combination with virtual screening to find novel hits. Deciding on the best pharmacophore model for virtual screening runs is often a tedious process relying on a large dataset of mostly artificially generated decoys and some truly active compounds. In addition to requiring large amounts of data, this evaluation process relies on the binary-classification of molecules into active and inactive ones. Molecules with similar activity values close to the cut-off are classified differently, although they demonstrate a quite similar experimental behaviour.

A quantitative pharmacophore model would be able to score other pharmacophore models and assign an estimated non-binary activity to these pharmacophores. The (biological) activity of pharmacophores can be interpreted as the expected activity of molecules matching such a pharmacophore. In the context of virtual screening, it is expected that the scored pharmacophore will retrieve molecules from a database with similar activity values. Therefore, the quantitative pharmacophore model can be easily applied as a ranking method to prioritise pharmacophore models generated by a researcher.

Despite the possible advantages hardly any research was done on quantitative pharmacophores and related methods. In contrast, QSAR applied on molecular structures has fostered plenty of research and a google-scholar search for the query “quantitative structure–activity relationships” yields close to 5 million results. Nevertheless, two commercially available tools have been released which are able to relate pharmacophores quantitatively to biological activity or other specified properties.

PHASE is a commercially available tool implemented in Maestro [9]. Besides pharmacophore perception, it allows for quantitative rationalisation of activity data based on 3D pharmacophore fields obtained from a set of ligands. Pharmacophores are created for each aligned ligand, whereas the alignment is not done automatically and needs to be considered by the user. The aligned pharmacophores are placed into a vectorised box, each voxel containing information about the value of the pharmacophore fields in that location. The box is used as input for a PLS-algorithm to regress the pharmacophore fields against a set of activity values. As output, the user gets a model displaying favourable as well as unfavourable regions contributing to the activity values. Additionally, the activity of new ligands can be predicted by feeding them to the model after alignment.

Even though PHASE provides one of two available QSAR methods for pharmacophores, it still relies on molecules as input for alignment and model building. Due to this shortcoming, apo-site derived pharmacophores or pharmacophores obtained from ligand-based modelling can only be predicted via workarounds. Therefore, pharmacophore QSAR within PHASE is similar to atom-based QSAR, except that an additional step for calculating the pharmacophore fields is carried out.

The second available method for pharmacophore QSAR is the Hypogen [12] algorithm implemented in the Catalyst program, which now is part of BioVia’s [13] Discovery Studio [14]. The Hypogen algorithm works utterly different from the PHASE algorithm, directly operating on pharmacophore features instead of using grids as a proxy. First, a subset of the most active compounds is chosen. All possible pharmacophore hypotheses from the two most active compounds are enumerated and must fit a minimum subset of the remaining compounds in the most active subset to be considered by the algorithm. From this generated set of pharmacophore hypotheses, the ones matching a group of inactive compounds are removed in a follow-up phase. In a third final phase, small perturbations are introduced to the remaining hypotheses, which are then scored based on the RMSE of predictions against the training set. The Hypogen refine algorithm extends this method by adding exclusion volumes and introducing another term in the loss function.

In contrast to PHASE, Hypogen is operating directly on pharmacophores without the need to provide the underlying molecules. However, a drawback of this method is still that it builds the quantitative models from a selected subset of highly active compounds. Even though refinement considers less-active compounds, we would expect predictions for pharmacophores obtained from less active molecules to be worse due to missing domain knowledge. After the model-building is done, no single quantitative model is selected, but a set of possible solutions is provided to the user, adding some ambiguity about the model’s quality.

Having in mind the potential advantages of a quantitative method that is based on pure pharmacophoric representations, we developed and herein present a novel approach for the generation of quantitative pharmacophore models. Based on a small dataset of molecules and/or pharmacophores, the proposed algorithm will first find a consensus pharmacophore (merged-pharmacophore) from all training samples. The input pharmacophores, or pharmacophores generated from the input molecules, will then be aligned to the merged-pharmacophore. For each aligned pharmacophore, information regarding its position relative to the merged-pharmacophore is extracted. This information is then used as input to a simple machine learning algorithm which derives a quantitative relationship of the merged-pharmacophores’ features with biological activities.

## Methods

### Datasets

#### Phase dataset

The dataset previously published by Debnath [15] et al. in 2002 was used as a benchmark datasets against the PHASE algorithm. Compounds were obtained in SMILES format, which served as input for the generation of 3D conformations. Conformations were generated using iConfGen [16] provided by LigandScout [17]. For all parameters default settings were used, and the maximum number of output conformations was set to 25. Training and test data were split according to the published results by Dixon et al. [8]. Molecule number 67 was removed from the dataset due to missing experimental activity data.

#### ChEMBL datasets

Datasets obtained from ChEMBL were used for cross-validation and assessment of the model’s robustness. The 23 most popular QSAR targets (Table 3), according to a list published by Cortés-Ciriano [18], were chosen for validation studies. The ChEMBL database was queried with the UniProtId of these 23 targets using the chembl-webresource-client [19]. Biological activity data ('standard_value'), in the following referred to as activity, was obtained for each compound and filtered by the following parameters:

- standard_type: ‘IC50’ or ‘Ki’.
- standard_units: ‘nM’.
- standard_relation: ‘ = ’.
- assay_type: ‘B’.
- target_organism: ‘Homo Sapiens’.

Activity readouts from biological assays depend heavily on the assay conditions and the environment. Due to the high experimental noise and the resulting inability to combine various assays, the datasets were separated by their ‘assay_chembl_id’. This resulted in datasets of ~ 20 to ~ 100 molecules.

The assay datasets were cleaned in a post-processing step to ensure the data was qualified for QSAR modelling. Datasets with less than three log-units difference between the minimum and maximum activity value were dismissed. Furthermore, to avoid overfitting and bias when training the ML models, the assay datasets were filtered by heterogeneity. Ideally, compound activities are distributed equally over the activity value range. To model data heterogeneity, the KL-divergence was calculated against a uniform distribution. The higher the KL values were, the more clustered the dataset was found to be. All datasets with KL values above a cut-off of 0.75 were discarded. The KL-divergence was calculated according to Eq. (1), whereas *P* and *Q* denote discrete uniform distributions over activity values for a given dataset. P represents the estimated uniform distribution of the datasets, Q resembles the reference uniform distribution over activity values, a being the minimum and b the maximum. P is estimated by binning the activity values into N (sample size) bins. Each P(x) is defined by the frequency of activity values in each bin x.

1 $$Eq 1: KL\left(P,Q\right)={\sum }_{x\epsilon X}P\left(x\right)*log\left(\frac{P\left(x\right)}{Q\left(x\right)}\right) $$

The ChEMBL datasets were split into training and validation data via a fivefold random split. This was done by applying a stratified K-fold procedure for quantitative data. Since stratified K-fold data splitting is usually used on classification datasets, a workaround was constructed where activity data was binned in K, five, classes [20]. In addition, a 20–80 split (20% training data, 80% validation data) was generated from the datasets as well as the standard 80–20 split (80% training data, 20% validation data). The 20–80 split aims to mimic a typical SAR setting experienced by a medicinal chemist, where only a limited amount of data is usually available. Training folds typically consisted of 10–15 samples.

For all datasets, 3D conformations were generated using LigandScout’s iConfGen. All parameters were left at their default values, and the maximum number of generated conformations was set to 25.

#### Baselines

Cross-validation studies were also carried out for two baseline methods using all ChEMBL datasets. One baseline model utilised the number of pharmacophore features per molecule as input to train a regression model. The second model was trained on physico-chemical descriptors of the input molecules. Only a limited number of descriptors was used due to the small dataset sizes. The following seven descriptors were calculated for the input molecules: number of H-Bond Donors/Acceptors, number of rotatable bonds, molecular weight, number of heavy atoms, cLogP [21], TPSA [22].

Additionally, the applicability domain of the baselines models was defined by calculating the min/max values [23] of the input vectors on the training fold. Test samples were deemed to be out-of-domain if their input vectors either fell below or exceeded the before determined min/max values, respectively.

### Machine learning model generation

To ensure a fair comparison, all baseline models were trained using the same machine learning algorithm and the same set of parameters as for the quantitative pharmacophores. Cross-validation studies were carried out using the random forest algorithm [24] implemented in the scikit-learn python package. The parameters ‘n_estimators’ and ‘max_depth’ were set to 10 and 3, respectively. The remaining parameters were kept at their defaults. No hyperparameter optimisation was performed for cross-validation studies due to the small datasets. Additional required parameters for the quantitative pharmacophores were set as follows (also the default parameters): fuzzy = True, weightType = distance, mostRigidTemplate = True.

Training the machine learning models of the quantitative pharmacophores on the PHASE dataset employed a protocol for hyperparameter optimisation. The following QPhAR specific parameters were subject to optimisation:

- weightType: [distance, nrOfFeatures, None].
- modelType: [random forests, ridge regression, PLS regression, PCA + ridge regression, PCA + linear regression].
- threshold: (1, 1.5, 2).

The parameters of the machine learning models were set to their default values, except for the random forest algorithm. Here the parameters ‘n_estimators’ and ‘max_depth’ were optimised by (10, 15, 20) and (2, 3), respectively.

### Quantitative pharmacophore algorithm

**Fig. 1:**
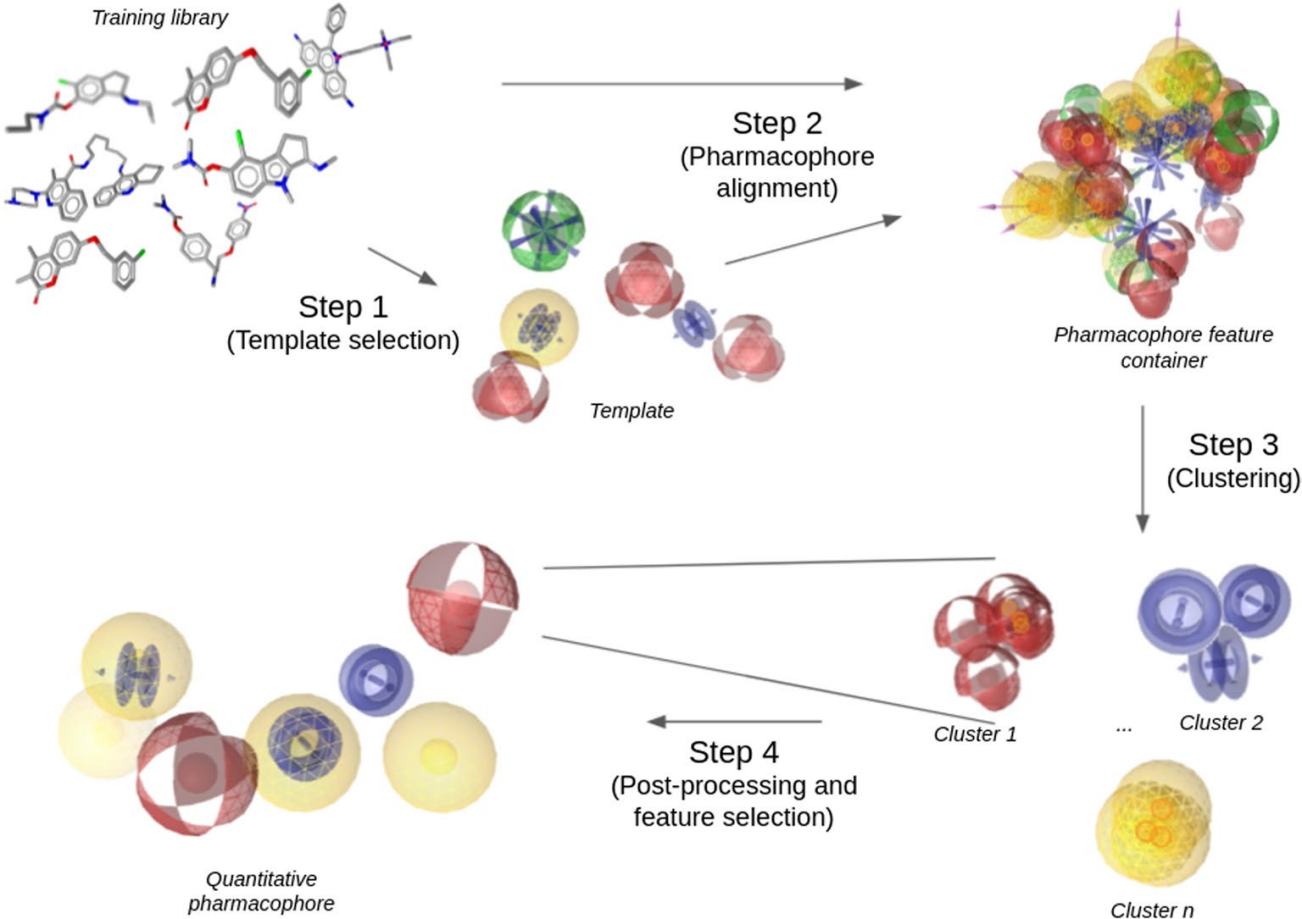
Schema of generating the quantitative pharmacophore model

**Fig. 2:**
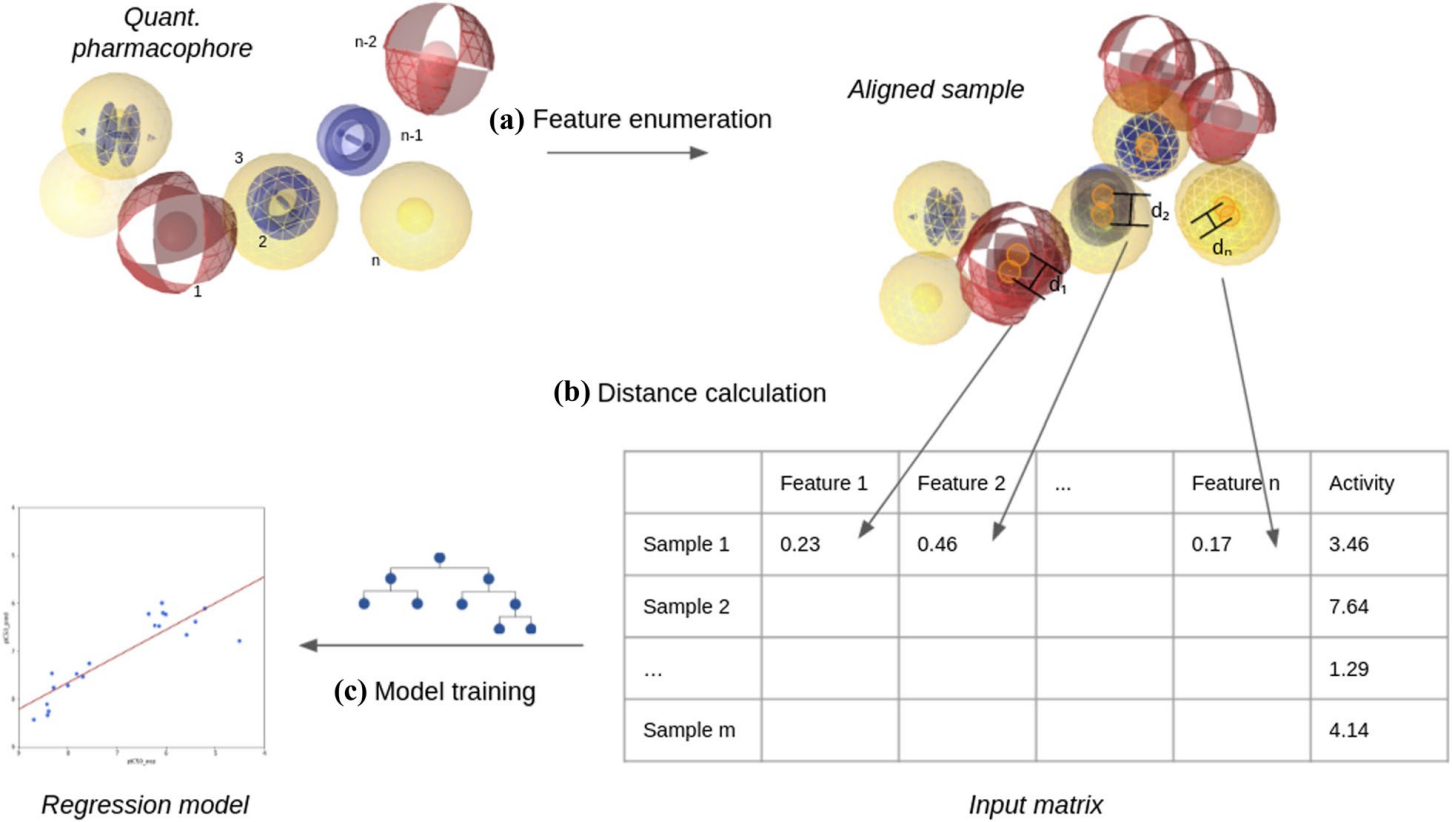
Feature extraction and ML-modeling (Step 5)

The generation of a quantitative pharmacophore model proceeds over five consecutive steps (Figs. 1, 2). At first, a template needs to be chosen (Step 1). The selected template could either be one of the training samples, such as the most rigid molecule, or any other pharmacophore or molecule the user deemed relevant. Selecting the template is highly important and can make or break the algorithm. If a poor template is chosen, the resulting alignment of the training samples might yield a quantitative pharmacophore model of low quality. In the second step, the training set is aligned to the selected template. Once all the samples are aligned, the pharmacophore features of the training samples are clustered (Step 3). In the following post-processing step (Step 4), representative features will be selected or generated for each found cluster. Furthermore, clusters with non-conclusive information get discarded, and only relevant clusters will be kept within the quantitative pharmacophore model. Finally, the remaining pharmacophore features from each cluster are used as input for training a regression machine learning model (Step 5).

#### Template selection

The quantitative pharmacophore algorithm provides two options for selecting a template: either a pharmacophore is given as input or a set of two molecules. A provided pharmacophore will be directly selected as the template. If two molecules are given, they will be aligned to each other via pharmacophore alignment that also regards the conformational flexibility of the molecules. Once the best alignment between the two molecules is found, a pharmacophore will be generated from the first molecule, which is then used as the template. A reasonable choice as the first molecule is to select the most rigid molecule from the training set.

#### Alignment

Once a template pharmacophore has been selected, the remaining training set will be aligned to the template using pharmacophore alignment and a greedy optimisation procedure to select the best fitting conformations. The aligned pharmacophores and their features are stored within a single pharmacophore data structure (container), creating a merged-pharmacophore-like outcome. Each pharmacophore feature is associated with the activity value of the parent pharmacophore. Directed pharmacophore features are converted to undirected spherical pharmacophore features due to the noise introduced by directed features. Each pharmacophore feature type is collected in a separate container, resulting in six distinct containers: hydrophobic (H), aromatic (AR), positive/negative ionisable (PI, NI), H-Bond donor/acceptor (HBD, HBA).

#### Clustering

A clustering algorithm then processes the collected pharmacophore features. The minimum distance hierarchical clustering algorithm is applied, whereas its cutoff is being treated as a hyperparameter. The default cutoff value is the radius of the pharmacophore features, 1.5 Å. A distance matrix containing the calculated euclidean distances between all features in the container is used as input for the clustering algorithm.

#### Post-processing

After successful clustering, two post-processing steps are applied. First, representative pharmacophore features are selected for each cluster and then clusters with non-conclusive activity data are removed, keeping only high impact pharmacophore features.

#### Representative feature selection

Ideally, each cluster can be represented by a single feature. If this is not possible, several features are placed to represent the cluster. The representative feature can either be one of the existing features in the cluster or be the product of merging all features in the cluster. A feature represents another feature if they overlap, meaning their distance is smaller than the radius of these pharmacophore features. The merged feature then inherits all activity values from the features it represents. Additionally, the number of included features is stored. Both these properties will be used in the second post-processing step.

The following options are considered to find representative features:

- Clusters containing a single feature: the cluster is already represented by the feature itself; therefore, no further modifications are necessary.
- Clusters containing multiple features: at first, each feature is probed as a representative feature. If any of the features within a cluster overlap with all other features, that feature is used as the cluster’s representative feature. Otherwise, a new feature is created. Three strategies are considered in order to determine the location of the new feature:
  - The feature is placed at the centroid of the cluster. If all features in the cluster overlap with the new feature, they are merged into the new feature and their properties assigned to the merged feature.
  - The new feature is placed in the centre of the cluster. The process of the centroid feature is repeated.
  - If all the aforementioned options fail to yield a representative pharmacophore feature, multiple features will be selected to represent the cluster. This process has no clear solution. Therefore, we apply a greedy iterative algorithm that maximises the number of represented features at each step. As long as non-merged features exist, the feature overlapping with most other features is selected as a representative feature. All properties of merged features are assigned to the selected feature. The just-merged features are then removed from the cluster. This process is repeated until all features in the cluster are assigned to a representative feature.

#### Removing ambiguous features

At this point, the quantitative pharmacophore consists of several features, each representing a cluster of pharmacophore features from the training set. However, some of these features will not add information to the final model. This is easy to imagine in the simple case of two molecules having the same scaffold but different residues and activities. One of the molecules shows high biological activity, whereas the other molecule is not active. A merged-pharmacophore will contain features representing the scaffold, as well as features representing the different residues. It is clear that the residues’ features can explain the activity of these molecules, and the scaffold does not contribute any information. This rationale is applied to the quantitative pharmacophore. Therefore, features containing activity values spanning more than half the distance of global maximum and minimum activity values are removed. They are considered non-conclusive regarding their activity and will not contribute relevant information to the quantitative model. Furthermore, pharmacophore features encountered only once are considered outliers and are removed from the quantitative pharmacophore model too due to missing validation of the feature’s importance. Finally, a cleaned, merged-pharmacophore is obtained, serving as a reference model in the machine learning procedure.

### ML model and weight selection

Given the training set and the reference merged-pharmacophore, input vectors are generated for each training sample. The vector has the size of the number of pharmacophore features in the reference pharmacophore. Each entry in the vector corresponds to one of the reference pharmacophore features. The vector entries are populated by the inverse euclidean distances between the features from the reference pharmacophore and the aligned training sample features. If these two features do not overlap, the entry is filled with a zero value. It follows that the quantitative pharmacophore algorithm does not consider features from training samples without corresponding features in the reference pharmacophore. This is on purpose and will be explained further in the “Applicability domain” section.

Optionally, weights can be added to the input vectors. If ‘weightType’ None is chosen, the distance values are converted to binary values, 1 for overlapping features, 0 for non-overlapping features. Specifying no weight type will keep the input vector as it is. The third option is to weigh the input vector by the number of features. Features consisting of a higher number of merged features are weighted heavier than features with fewer data.

Once input features are generated for each aligned training sample, a regression machine learning model is trained on the input vectors and the activity data. The type of the machine learning model is a hyperparameter of the quantitative pharmacophore algorithm. However, it is recommended to use simple algorithms to avoid overfitting. A linear regression model, a ridge regression model, and a heavily restricted random forest model have been applied in this study.

The current version of the algorithm has been implemented in Python using the Chemical Data Processing Toolkit [25] (CDPKit) for the representation and processing of molecule and pharmacophore data. Machine learning models were trained using the scikit-learn package [26]. The code and all datasets are available at https://github.com/StefanKohlbacher/QuantPharmacophore.

### Applicability domain

Applicability domains are an essential part of machine learning algorithms [27, 28]. Samples outside the domain of the training data cannot be predicted with high confidence. Moreover, the predictions could be random values not reflecting the true values at all. Therefore, the applicability domain for quantitative pharmacophore models gets defined by two factors. First, suppose a new sample, molecule or pharmacophore cannot be aligned to the template of the quantitative pharmacophore due to missing features or dissimilarities. In that case, the sample is deemed as out-of-domain and will not be predicted by the model. Second, due to the removal of features with non-conclusive activity values, some features in the query sample may not be matched with the quantitative pharmacophore. Furthermore, features not overlapping with any of the model’s features will not contribute information to the input vector. Even though the model is missing information in such cases, these non-overlapping features must not be included in the input vector due to missing training data in these regions. Therefore, not the entire sample is out-of-domain, but only certain features within the sample. Features out-of-domain are not included during inference.

## Results and discussion

The quantitative pharmacophore model is obtained by first creating a merged-pharmacophore. Data from the merged-pharmacophore and the training set is then used to fit a machine-learning model. Training of the ML-model is carried out with the same dataset as the merged-pharmacophore was created from. Therefore, the dataset is required to have known activity values for each sample. Creating a merged-pharmacophore as the underlying model has several advantages. For one, it keeps the model explainable and straightforward, unlike many other black-box ML algorithms. Second, due to the familiar merged-pharmacophore concept and representation, the model can quickly be adopted by scientists already familiar with such tools. The steep learning-curve allows a medicinal chemist to iterate through ideas quickly.

As mentioned before, there are only a few tools currently available to the scientific community allowing scientists to perform QSAR from pharmacophores. Here we do not directly compare against these methods since the quantitative method described in this paper expands to domains not accessible by previous algorithms. Nevertheless, we show that the quantitative pharmacophore performs similar to the PHASE algorithm on molecule datasets. Furthermore, based on a broad set of commonly used protein-targets for QSAR, we prove that the method shows robust performance over a wide variety of datasets.

### PHASE vs. QPhAR

**Fig. 3:**
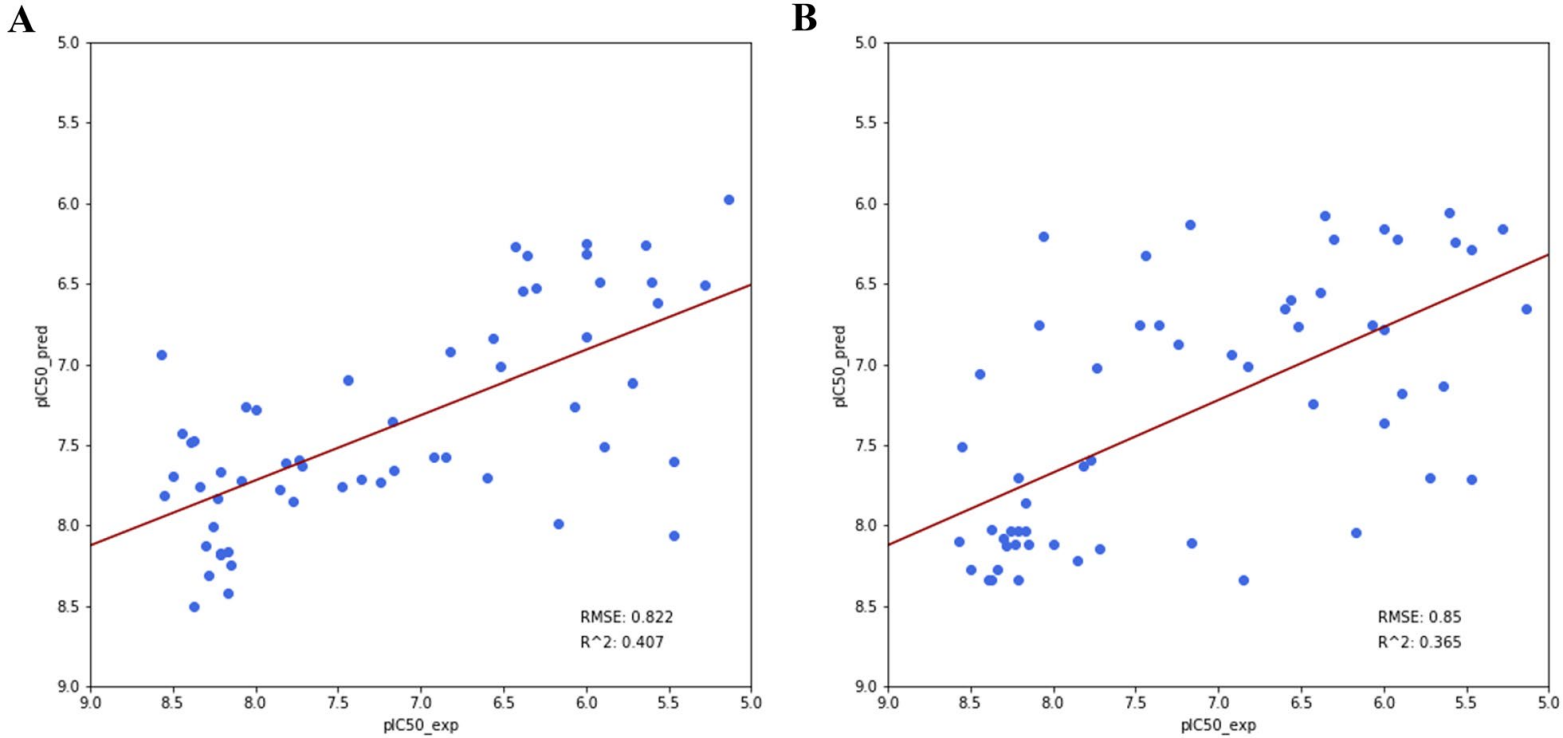
Test set performance of PHASE (**A**) and the quantitative pharmacophore model (**B**)

The paper published by Dixon et al. [8] in 2006 describing the PHASE algorithm compares its method against an even earlier published paper [15] using the Hypogen algorithm to predict the activities of a dataset. The quantitative pharmacophore was trained on the same training set, 20 samples, as described in the paper by Debnath et al. [15]. It was then evaluated on the holdout test set containing 57 molecules (originally 58, but one sample had no reported activity value). The reported RMSE and R^2^ values on the test set of the PHASE algorithm were 0.822 and 0.407 (Fig. 3A), respectively. In contrast, the quantitative pharmacophore model could achieve an RMSE of 0.85 and an R^2^ of 0.365 (Fig. 3B). These two models are comparable to each other and are expected to yield similar results on new datasets. A student t-Test has further validated the fact that the models perform on par. The student t-Test was performed using scipy’s implementation for related samples, as is the case here for predictions from the same test set. The t-Test resulted in a p-value of 0.29. Therefore, the null-hypothesis that the two models perform differently cannot be rejected. To reject the null-hypothesis a p-value of at least 0.05 or lower should be achieved. Figure 5 shows the mean RMSE values of bootstrapping the testset for both the PHASE and the QPhAR models. The 95% confidence intervals obtained from bootstrapping show a significant overlap between the two methods (Fig. 5 Appendix). The distribution of prediction errors is plotted in Fig. 6 (Appendix) for both models. An interesting fact that can be observed is the slightly lower median of the quantitative pharmacophore model compared to PHASE. Although, this does not indicate superiority over PHASE due to the previously stated equality of the two models.

**Fig. 4:**
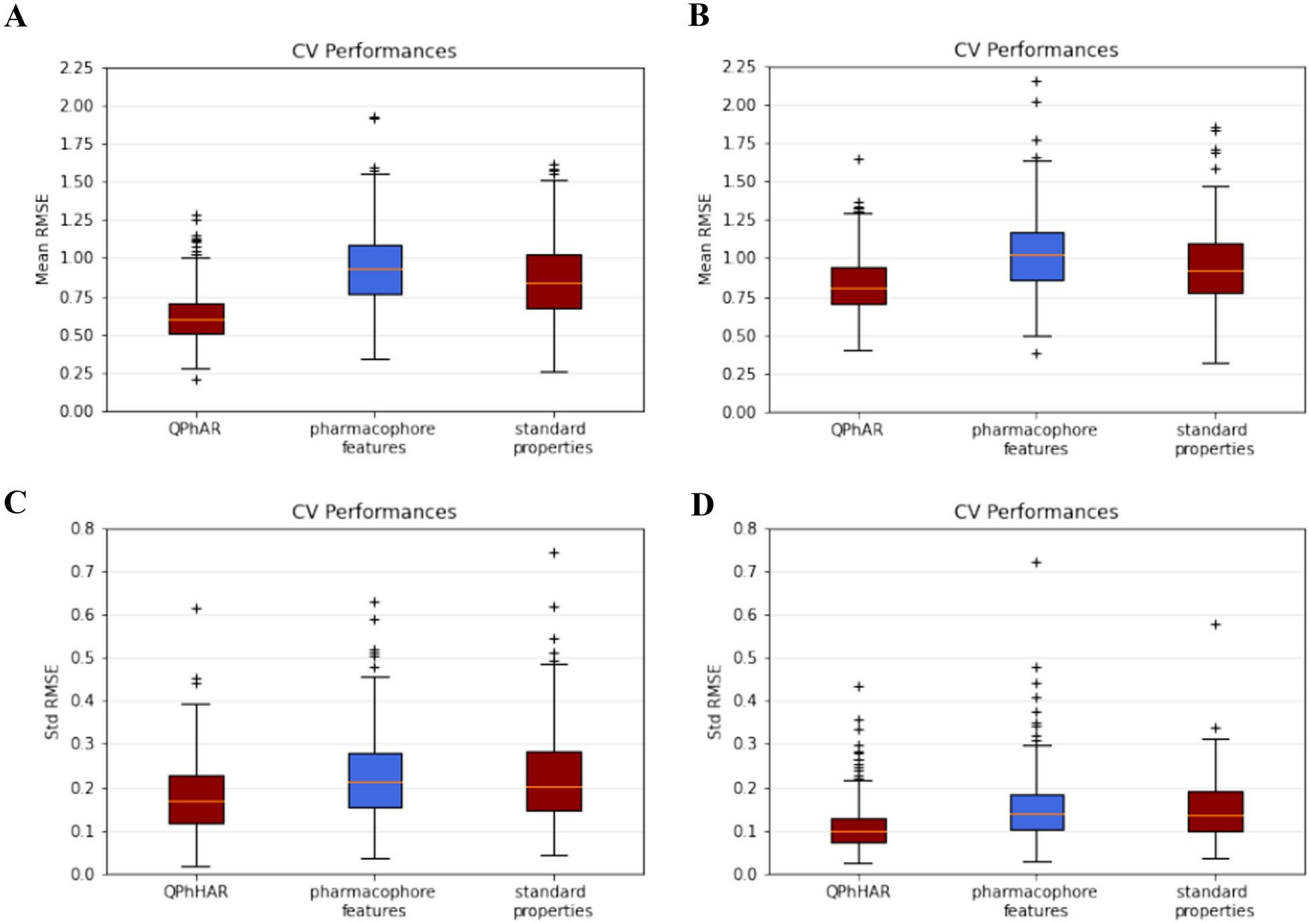
Aggregated CV performance over > 250 datasets from quantitative pharmacophores and two baseline methods. **A** Mean RMSE values of models in 80–20 split. **B** Mean RMSE values of models in 20–80 split. **C** Mean standard deviation of models in 80–20 split. **D** Mean standard deviation of models in 20–80 split

However, it is important to keep in mind that both models were trained and evaluated on molecules, which is not the main focus of the method described here. Prediction of pharmacophores not obtained from molecules is one of the unique strengths of the quantitative pharmacophores. Alignment and prediction of such pharmacophores has not been possible before and prediction of pharmacophores with other methods still relied on molecules for alignment. Therefore, the method described here does not aim to compete against previous algorithms but rather expands the toolbox available to researchers while still providing similar quality results as state-of-the-art methods.

### Cross-validation

Besides comparing our method against existing methods, CV was carried out on more than 250 distinct datasets to test the quantitative pharmacophores’ general applicability across a wide range of datasets. All training-validation runs used default parameters to gauge the quantitative pharmacophores’ effectiveness when used out-of-the box. Simple baseline models were built to demonstrate superior behaviour over standard methods. As baselines, the number of pharmacophore features in the training set was regressed against the activity endpoint. Furthermore, a set of simple physico-chemical properties was calculated and used as a second baseline model input. To ensure a fair comparison, all baseline models used the same machine learning algorithm with default parameters as the quantitative pharmacophores. Cross-validation runs were evaluated by calculating the mean RMSE and the standard deviation of the RMSE over the five individual runs (Fig. 4). Additionally, the applicability domain has been investigated for the test set samples. Samples deemed out-of-domain for baseline models were further analysed and compared against in-domain predictions from the quantitative pharmacophores. No sample was found to be out-of-domain for the quantitative pharmacophores.

**Fig. 7:**
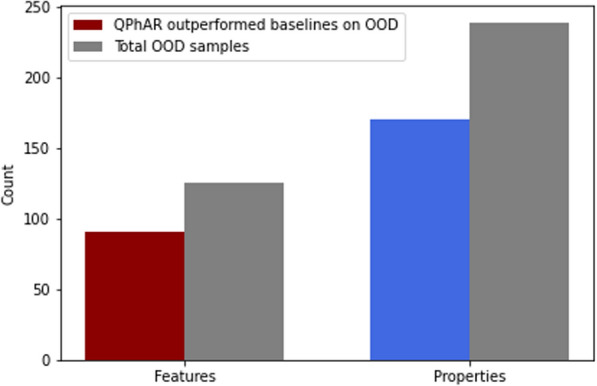
Number of times QPhAR outperformed baselines for out-of-domain (OOD) samples (80–20 cv split)

Mean RMSE values of the CV (80–20 split; Fig. 4A) range from 0.20 to 1.27, with an average over all datasets of 0.62. Generally, an average mean RMSE of 0.62 over all datasets is perceived as quite good, considering that lower RMSE values are a strong indication of modelling the experimental noise in the datasets. With that in mind, the small number of datasets with mean RMSE values of 0.20 from CV are very likely to overfit. On the other hand, the worst RMSE of 1.27 was obtained with default parameters and in depth analysis and model optimisation are likely to sharply increase the model’s performance. Along with mean RMSE values, the standard deviation of the model’s CV performance was calculated, which on average, was 0.18 across the five folds CV. Further emphasis in the analysis was put on the applicability domain. The applicability domains for the baselines were defined as described in the “Methods” section. The applicability domain for the quantitative pharmacophore is defined by a possible alignment of the test samples to the model. If no alignment with the model can be found, the sample is out-of-domain. During CV runs, no test samples were found to be out-of-domain for the quantitative pharmacophore model. However, out of 8520 test samples from all CV runs, around $${\raise0.7ex\hbox{$1$} \!\mathord{\left/ {\vphantom {1 8}}\right.\kern-\nulldelimiterspace} \!\lower0.7ex\hbox{$8$}}$$ (901) of the test samples were found to be out-of-domain for the physico-chemical properties baseline and ~ 3% (244) for the pharmacophore features baseline. Predictions of these samples were made nevertheless and then further analysed in comparison with the quantitative pharmacophores. Our model could improve on these predictions in 71%, and 72% of all out-of-domain samples for the properties and features baseline, respectively. Figure 7 (Appendix) shows the number of times the quantitative pharmacophore model yielded better predictions than the baselines, when the test-samples were found to be out-of-domain. For a summary of the quantitative pharmacophores and baselines 80–20 CV results see Table 1.

**Table 1:** Results for 80–20 CV of quantitative pharmacophores and baselines

	Quantitative pharmacophores	Features baseline	Properties baseline
RMSE (mean)	0.62	0.95	0.86
RMSE (standard deviation)	0.18	0.24	0.24
RMSE (minimum)	0.2	0.34	0.26
RMSE (maximum)	1.27	1.92	1.61
Samples out of domain	0	244 (2.9%)	901 (10.1%)

Evaluation of the 20–80 split (Fig. 4B) yielded an average RMSE of 0.83 over all datasets, with a minimum RMSE of 0.41 and a maximum of 1.65. As expected, these models’ performance is not as high as the average performance from models trained on the 80–20 split. These results agree with the general notion that more data will improve a machine learning model’s quality. However, considering that the training sets contained only 10–15 samples, the model’s performances are respectably high. A valid concern often raised with such low training set sizes is the potential overfitting of the models. Here, we can exclude overfitting since the trained models were evaluated on validation sets four times larger than the training sets. If models would overfit, the performance on the validations sets would be considerably worse and therefore not be in agreement with obtained results. Furthermore, the standard deviation over all datasets of the 20–80 CV is much lower than the 80–20 CV, Fig. 4D, C, respectively. The low standard deviation further strengthens the point that the models are not overfitting any of the CV splits. Nevertheless, the small standard deviation compared to the 80–20 split is surprising since smaller datasets are expected to increase the model’s performance variance. Achieving a smaller variance during CV with smaller datasets further boosts confidence in robust quantitative pharmacophores.

**Fig. 8:**
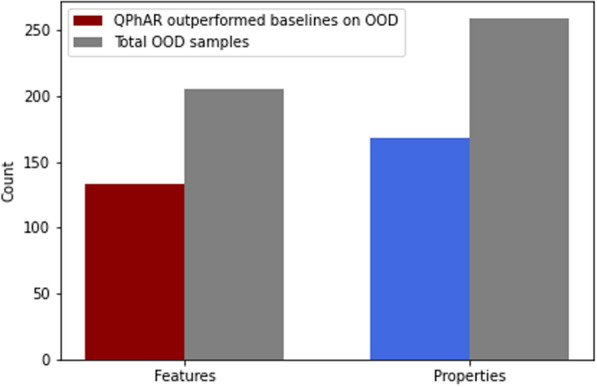
Number of times QPhAR outperformed baselines for out-of-domain (OOD) samples (20–80 cv split)

As for the 80–20 split, additional analysis was performed for samples classified as out-of-domain. Due to the small training sets, considerably more samples from the test set were found to be out-of-domain for the baseline models as with the 80–20 split. Out of 19,805 test samples in total, > 23% (4602) were deemed as out-of-domain for the properties baseline and ~ 7% (1399) for the features baseline. No test sample was found to be out of domain for the quantitative pharmacophores. 65% of the time predictions were improved by the quantitative pharmacophores (Fig. 8 Appendix). For a summary of the quantitative pharmacophores and baselines 20–80 CV results see Table 2. For a list of all target-proteins used in the CV studies see Table 3.

**Table 2:** Results for 20–80 CV of quantitative pharmacophores and baselines

	Quantitative pharmacophores	Features baseline	Properties baseline
RMSE (mean)	0.83	1.04	0.94
RMSE (standard deviation)	0.19	0.25	0.25
RMSE (minimum)	0.41	0.39	0.32
RMSE (maximum)	1.65	2.15	1.86
Samples out of domain	0	1399 (7.1%)	4602 (23.2%)

In direct comparison to the baselines, the quantitative pharmacophore model was superior in ~ 9/10 cases measured by the RMSE of CV (80–20 split as well as 20–80 split). In the 80–20 split CV, the quantitative pharmacophore could improve the mean RMSE by 34% over the pharmacophore features baseline and 27% over the physico-chemical properties baseline. Similar results can be seen on the 20–80 split, where 20% and 12% improvement was achieved, respectively.

## Conclusion

Pharmacophores are widely applied in a qualitative manner for hit identification in virtual screening experiments and hardly any information can be found on the quantitative use of pharmacophore models. PHASE and Hypogen, only accessible in commercial packages, currently provide the only two algorithms which allow for quantitative insights on pharmacophore models. Targeting their drawbacks, such as alignment, the requirement of molecules for training, and user-friendliness, we present a novel quantitative pharmacophore generation algorithm for QSAR studies. The algorithm first creates a merged-pharmacophore from a given set of molecules and/or pharmacophores. Information obtained from aligning the training set to the merged-pharmacophore is then used to train a machine-learning model. We performed extensive cross-validation on a large variety of datasets and could show that quantitative pharmacophore models generated by our methods generalise well to many different datasets even without performing hyperparameter optimisation. The trained models achieved a mean RMSE of 0.61 during CV over > 250 datasets. The datasets used for CV resemble sizes typically encountered in SAR settings by medicinal chemists. We could also demonstrate the robustness of our algorithm which is insensitive to small perturbations during training by achieving small variance in RMSE over fivefold CV. Furthermore, on more than 90% of datasets, the generated quantitative pharmacophore models outperformed tested baselines, thus making our method a reasonable first approach for any researcher looking to get quantitative SAR insights on his data.

### Supplementary Information

**Additional file 1.** Metadata of all datasets used for CV studies with 20–80 split.

**Additional file 2.** Metadata of all datasets used for CV studies with 80–20 split.

## Figures and Tables

**Fig. 5:**
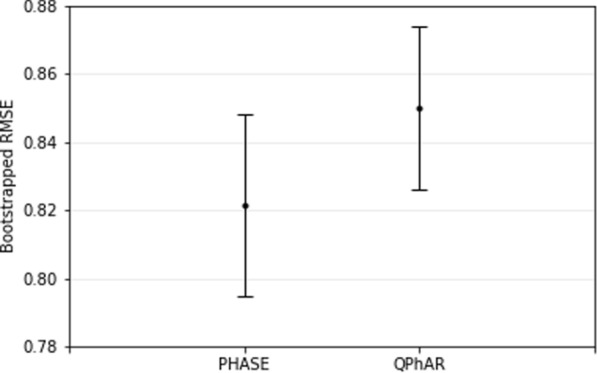
RMSE values of retrospective bootstrapping including 95% confidence intervals

**Fig. 6:**
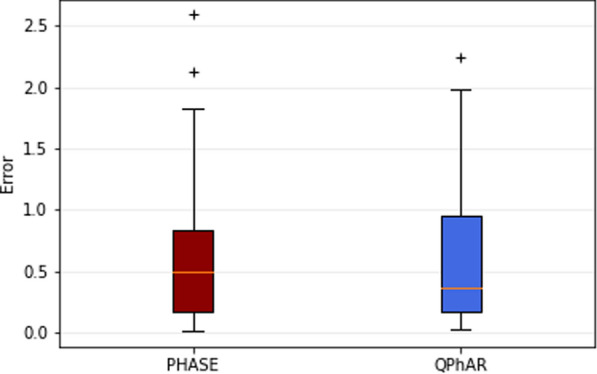
Error distribution of predictions from PHASE and QPhAR

## Data Availability

The datasets supporting the conclusions of this article are available in the Github repository https://github.com/StefanKohlbacher/QuantPharmacophore. Metadata of all datasets used for CV studies are provided in Additional File 1 and Additional File 2 for CV-split 20-80 and CV-split 80-20, respectively.

## Appendix

For a list of target-proteins used for CV studies see Table 3.

**Table 3:** Target list used for model validation

Target	UniProt ID	CHEMBL ID
Alpha-2a adrenergic receptor	P08913	CHEMBL1867
Tyrosine-protein kinase ABL	P00519	CHEMBL1862
Acetylcholinesterase	P22303	CHEMBL220
Androgen receptor	P10275	CHEMBL1871
Serine/threonine-protein kinase Aurora-A	O14965	CHEMBL4722
Serine/threonine-protein kinase B-raf	P15056	CHEMBL5145
Cannabinoid CB1 receptor	P21554	CHEMBL218
Carbonic anhydrase II	P00918	CHEMBL205
Caspase-3	P42574	CHEMBL2334
Thrombin	P00734	CHEMBL204
Cyclooxygenase-1	P23219	CHEMBL221
Cyclooxygenase-2	P35354	CHEMBL230
Dihydrofolate reductase	P00374	CHEMBL202
Dopamin D2 receptor	P14416	CHEMBL217
Norepinephrine transporter	P23975	CHEMBL222
Epidermal growth factor receptor erbB1	P00533	CHEMBL203
Estrogen receptor alpha	P03372	CHEMBL206
Glucocorticoid receptor	P04150	CHEMBL2034
Glycogen synthase kinase-3 beta	P49841	CHEMBL262
HERG	Q12809	CHEMBL240
Tyrosine-protein kinase JAK2	O60674	CHEMBL2971
Tyrosine-protein kinase LCK	P06239	CHEMBL258
Monoamine oxidase A	P21397	CHEMBL1951
Mu opioid receptor	P35372	CHEMBL233
Vanilloid receptor	Q8NER1	CHEMBL4794

## Quantitative pharmacophore model applied on the dataset from Debnath et al.

A quantitative pharmacophore model was trained on the same dataset as by Dixon et al. for the PHASE algorithm. The hyper-parameters of the best model were as follows (Table 4):

**Table 4:** Hyperparameters of trained quantitative pharmacophore model on datasets from Debnath et al.

Parameter	Value
Fuzzy	True
Modeltype	RandomForest
Threshold	1
Weighttype	distance
maxDepth (of ML-Model)	3
nEstimators (of ML-Model)	10

The predictions of the model on the training and test set can be found in the following Tables 5 and 6, respectively.

**Table 5:** Predictions on test set of quantitative pharmacophore model

Index	pIC50 exp	pIC50 pred	Index	pIC50 exp	pIC50 pred
0	5.64	7.13	35	8.57	8.10
1	6.6	6.66	37	7.74	7.02
2	6.07	6.75	38	6	6.78
3	6.43	7.24	42	8.17	7.86
4	6.92	6.94	43	6.3	6.22
5	6.52	6.77	44	5.47	7.71
6	6.56	6.60	45	7.17	6.13
7	7.16	8.11	47	6.36	6.08
8	7.77	7.59	48	8.44	7.06
9	7.72	8.14	49	8.15	8.11
10	6	7.36	52	6.85	8.34
11	5.72	7.70	53	8.23	8.11
12	8.09	6.75	54	5.47	6.28
13	8.21	8.04	55	8.37	8.02
14	7.44	6.33	59	7.82	7.63
15	7.48	6.75	61	8.21	7.70
18	8.39	8.34	62	5.57	6.24
19	6.82	7.01	63	8.5	8.28
20	8	8.11	64	8.21	8.34
21	6.17	8.04	65	5.89	7.18
22	7.36	6.75	66	5.6	6.06
24	8.3	8.08	67	8.37	8.34
25	8.55	7.51	68	6	6.16
26	8.06	6.20	69	6.38	6.55
27	8.28	8.13	70	7.24	6.88
28	5.92	6.22	71	7.85	8.22
30	8.26	8.04	72	8.34	8.28
32	8.17	8.04	76	5.14	6.65
34	5.28	6.16

**Table 6:** Predictions of quantitative model training set

Index	pIC50 exp	pIC50 pred	Index	pIC50 exp	pIC50 pred
16	7.82	7.48	46	8.29	7.76
17	7.57	7.25	50	5.41	6.38
23	6.09	5.98	51	6.23	6.46
29	7.70	7.53	56	6.07	6.19
31	8.33	7.46	57	6.00	6.23
33	8.00	7.71	58	6.15	6.47
36	4.51	6.78	60	8.40	8.25
39	5.21	6.10	73	5.59	6.65
40	8.43	8.10	74	8.70	8.42
41	8.42	8.34	75	6.36	6.22

Metadata such as dataset sizes, span of activity values, etc. for all datasets used for CV is provided in Additional file 1 (CV 20–80 split) and Additional file 2 (CV 80–20 split).

## PHASE vs QPhAR additional plots

Figures 5 and 6 display the bootstrapped confidence intervals as well as the error distributions of the predictions from the PHASE and QPhAR models.

## Cross-validation additional plots

Figures 7 and 8 display the frequency of the quant. pharmacophore models outperforming OOD predictions of the baselines. 
